# RETRACTION: Impact of Obese Patients in Ovarian Cancer Surgery on Postoperative Wound Complications: A Meta‐Analysis

**DOI:** 10.1111/iwj.70350

**Published:** 2025-03-17

## Abstract

**RETRACTION:** HuangX. , DuS. , WangQ. , YangC. , LiuX. , ChenK. , ZhaoY. , HeN. , WangH. , “Impact of Obese Patients in Ovarian Cancer Surgery on Postoperative Wound Complications: A Meta‐Analysis,” International Wound Journal 21, no. 4 (2024): e14439, 10.1111/iwj.14439.38064172 PMC10957721

The above article, published online on 08 December 2023, in Wiley Online Library (http://onlinelibrary.wiley.com/), has been retracted by agreement between the journal Editor in Chief, Professor Keith Harding; and John Wiley & Sons Ltd. Following an investigation by the publisher, all parties have concluded that this article was accepted solely on the basis of a compromised peer review process. The editors have therefore decided to retract the article. The authors did not respond to our notice regarding the retraction.

**RETRACTION:** X. Huang , S. Du , Q. Wang , C. Yang , X. Liu , K. Chen , Y. Zhao , N. He , H. Wang , “Impact of Obese Patients in Ovarian Cancer Surgery on Postoperative Wound Complications: A Meta‐Analysis,” International Wound Journal 21, no. 4 (2024): e14439, 10.1111/iwj.14439.38064172 PMC10957721

The above article, published online on 08 December 2023, in Wiley Online Library (http://onlinelibrary.wiley.com/), has been retracted by agreement between the journal Editor in Chief, Professor Keith Harding; and John Wiley & Sons Ltd. Following an investigation by the publisher, all parties have concluded that this article was accepted solely on the basis of a compromised peer review process. The editors have therefore decided to retract the article. The authors did not respond to our notice regarding the retraction.

